# Long-term outcomes after unilateral salpingo-oophorectomy: A registry-based retrospective cohort study

**DOI:** 10.1371/journal.pmed.1004639

**Published:** 2025-07-07

**Authors:** Huan Yi, Naiqi Zhang, Jan Sundquist, Kristina Sundquist, Xiangqin Zheng, Jianguang Ji

**Affiliations:** 1 Department of Gynecologic Oncology, Fujian Maternity and Child Health Hospital College of Clinical Medicine for Obstetrics & Gynecology and Pediatrics, Fujian Medical University, Fuzhou, Fujian, China; 2 National Key Gynecology Clinical Specialty Construction Institution of China, Fujian Provincial Key Gynecology Clinical Specialty, Fujian Maternity and Child Health Hospital, Affiliated Hospital of Fujian Medical University, Fuzhou, Fujian, China; 3 Center for Primary Health Care Research, Department of Clinical Sciences Malmö, Lund University, Malmö, Sweden; 4 Center for Community-based Healthcare Research and Education (CoHRE), Department of Functional Pathology, School of Medicine, Shimane University, Shimane, Japan; 5 University Clinic Primary Care Skåne, Region Skåne, Sweden; 6 Faculty of Health Sciences, University of Macau, Macau SAR, China; 7 Cancer Center, Faculty of Health Sciences, University of Macau, Macau SAR, China; Makerere University College of Health Sciences, UGANDA

## Abstract

**Background:**

Opportunistic bilateral salpingo-oophorectomy (BSO) is recommended in women who have undergone a hysterectomy due to gynecological carcinomas and/or in women with genetic indications, especially for women who do not intend to conceive. However, there is ongoing debate about whether BSO should be recommended in premenopausal women, due to the early cessation of estradiol because of BSO which is linked to several health concerns, including coronary artery disease (CAD) and osteoporosis. This study aims to explore whether ovarian cancer can be prevented by unilateral salpingo-oophorectomy (USO) while not affecting the long-term risk of CAD and osteoporosis.

**Methods and findings:**

By accessing the Swedish national registries, this retrospective cohort study included 42,306 women who underwent USO between 1993 and 2018 before the age of 50 years. These women were randomly matched with 211,530 women who had not undergone USO using a propensity score matching approach to ensure comparability between the groups. Follow-up started on the date of USO operation and continued until the earliest occurrence of the following events: diagnosis of specific outcomes of interest, death from any cause, or the end of the study period (31st December 2018). Cox regression models were used to calculate hazard ratios (HRs) and 95% confidence intervals (CIs) of overall and histology-specific ovarian cancer, CAD, and osteoporosis associated with USO. After a median follow-up of 13 years, our analyses revealed that USO was not associated with subsequent risk of CAD (HR = 1.02, 95% CI [0.95, 1.09]) and osteoporosis (HR = 1.06, 95% CI [0.98, 1.16]). However, USO was significantly associated with a reduced risk of high-grade serous ovarian carcinoma (HR = 0.64, 95% CI [0.45, 0.92]). No differences were found when the analyses were stratified by hysterectomy. The main limitation of the study was that some confounding factors, such as BRCA1/2 pathological mutant status, were not available in our database.

**Conclusions:**

Our study suggests that USO reduces the risk of HGSCs but was not associated with CAD or osteoporosis after a median 13-year follow-up. These results suggest that USO may be a safer option than BSO for lowering ovarian cancer risk in premenopausal women, as it could avoid the negative health effects of early menopause.

## Author summary

**Why was this study done?:**

- Bilateral salpingo-oophorectomy is associated with a lower risk of ovarian cancer but a higher risk of coronary artery disease (CAD) and osteoporosis.
- The associations between opportunistic unilateral salpingo-oophorectomy (USO) performed in women before the age of 50 and the long-term risk of histologically specific ovarian cancer, CAD, and osteoporosis remain unclear.

**What did the researchers do and find?:**

- This nationwide cohort study in Sweden revealed that USO before the age of 50 was not associated with the subsequent risk of CAD and osteoporosis.
- Women who underwent USO had a 36% lower risk of high-grade serous ovarian cancer.

**What do these findings mean?:**

- Opportunistic USO may serve as an alternative to BSO and prophylactic bilateral salpingectomy for reducing the risk of high-grade serous ovarian cancer.
- A key advantage of USO over BSO is that USO does not increase the subsequent risk of CAD and osteoporosis, which are commonly associated with BSO, especially for women who underwent operation before the age of 50.
- A key limitation of this study is that we couldn’t account for certain other factors, like whether patients had harmful BRCA1 or BRCA2 gene changes, because this information wasn’t in our records.

## Abstract 3

In a registry-based retrospective cohort study, Huan Yi and colleagues investigated the long-term outcomes of unilateral salpingo-oophorectomy (USO), and find that USO is associated with a reduced risk of ovarian carcinoma, but not with increased risk of coronary artery disease and osteoporosis.

## Introduction

Globally, ovarian cancer ranks third in terms of incidence and second in terms of mortality among malignant tumors of the female reproductive system [1]. The latest cancer statistics indicate that 19,710 new cases and 13,270 deaths were reported in the United States in 2023 [2]. Approximately 70% of patients with ovarian cancer are diagnosed at an advanced stage due to the lack of validated early screening tools. Additionally, the highly heterogeneous nature of the tumor cells, which do not phenotypically resemble any normal cells in the ovary, results in a five-year survival rate of less than 47% [3,4]. Therefore, it is imperative and crucial to assess the efficacy and appropriateness of prophylactic and opportunistic surgeries that aim to prevent ovarian cancer.

Epithelial ovarian cancer is the most common pathologic type, accounting for about 85% of cases [5]. Epithelial ovarian cancer can be classified into five major genetically and clinically distinct histological subtypes: high- and low-grade serous, mucinous, endometrioid, and clear-cell ovarian carcinomas. Among them, high-grade serous carcinomas (HGSCs) are the most lethal subtypes [6]. The ovarian surface epithelium was previously considered to be the primary origin of HGSCs [7]. However, a series of epidemiological, clinical, pathological, and molecular data [8–11] suggest that HGSCs predominantly originate from serous tubal intraepithelial carcinoma and microinvasive carcinoma of the fimbriae of the fallopian tube and its precancerous lesions [12,13]. This fallopian tube origin is further supported by the high-frequency TP53 mutations found in newly identified HGSCs, which are similar to those in fallopian tube cells [14].

Bilateral salpingo-oophorectomy (BSO) is recommended to be performed in premenopausal women without fertility desire in case of gynecological malignancies or a genetic predisposition to ovarian cancer [15]. As for non-malignant indications at the time of hysterectomy, it remains a matter of debate as to whether to perform opportunistic BSO [16–18]. Among U.S. women who underwent opportunistic BSO along with hysterectomy for ovarian cancer prevention, an estimated 23% of them were aged 40–44 years and 45% of them were aged 45–49 years, even though they were at average risk of developing ovarian cancer [19]. However, BSO performed in premenopausal women leads to an abrupt and substantial decline in estrogen, progesterone, and androgen levels, which can trigger premature menopause, and adversely affect coronary artery disease (CAD) and bone health, which have been extensively documented [20,21]. Unilateral salpingo-oophorectomy (USO), preserving one ovary to maintain female reproductive and endocrine function, is mainly performed for women who have endometriosis, benign neoplasms, inflammation and unilateral stage I tumors (stage 1A) and/or low risk tumors, such as low malignant potential lesions, early-stage invasive epithelial tumors, mucinous tumors, malignant germ cell tumors, or malignant sex cord-stromal tumors who wish to preserve fertility [15]. However, it is still unknown whether USO can prevent the development of ovarian cancer and without leading to long-term risks of CAD and osteoporosis. In this nationwide cohort study, we aimed to explore the long-term health outcomes among women who received USO, with a main focus on overall and histologic-specific ovarian cancer, CAD, and osteoporosis.

## Methods

### Data sources

**Fig 1:**
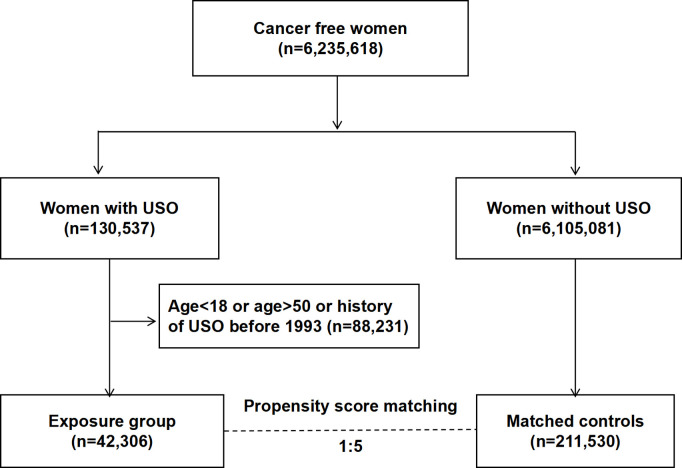
Flow chart of women involved in this nationwide cohort study in Sweden. The propensity score was calculated based on the date of birth, birth country, education level, region of residence, income, obesity, COPD, CCI, history of endometriosis, age at first delivery, and family history of breast or ovarian cancer. Abbreviations: USO, unilateral salpingo-oophorectomy; CCI, Charlson comorbidity index; COPD, chronic obstructive pulmonary disease.

This nationwide retrospective cohort study was approved by the Ethics Committee at Lund University, Sweden (Dnr 2012/795 and later amendments). Informed consent was waived due to the use of de-identified data. The study population comprised more than 6 million cancer-free females registered in Sweden as of January 1993, identified from the Swedish Total Population Register containing detailed demographic data on almost 100% of Sweden’s residents [22]. By linking to the Swedish Patient Register, we identified 42,306 women aged 18–50 years who underwent USO from January 1993 to June 2018. To ensure that all USO cases represented first-time procedures, women with a history of USO before the cohort entry date (January 1, 1993) were excluded from the study (Fig 1). The date of the USO operation was used as the index date. For each patient with USO, five women who had not undergone USO by the date of the procedure in the corresponding case were randomly selected from the source population and matched with the case by propensity score which was calculated based on the date of birth, birth country, education level, region of residence, income, obesity, chronic obstructive pulmonary disease (COPD), Charlson comorbidity index (CCI), history of endometriosis, age at first delivery, and family history of breast or ovarian cancer. The index date of the control patients was the same as that of the corresponding patient with USO (the date of operation) [23]. To further strengthen the robustness of our findings, we have also identified women with BSO from the National Patient Register and compared the incidence of CAD and osteoporosis with that of women with USO. This study is reported following the REporting of studies Conducted using Observational Routinely collected health Data (RECORD) guidelines (S1 RECORD Checklist) [24].

### Assessment of covariates

By retrieving data from the National Patient Register, Statistics Sweden’s Total Population Register and Population Housing Census, and Swedish Multiple Generation Register, we extracted information on potential confounding factors, including date of birth, birth country (Sweden or others), highest education (1–9, 10–11, and ≥12 years) [25], region of residence (big city, south, north), income (lowest, middle-low, middle-high, highest), obesity (identified from the National Patient Register using International Classification of Diseases (ICD) code E66, yes/no), COPD (yes/no) as a proxy for smoking, and CCI (0, 1, 2, and ≥3), history of endometriosis (yes/no, the main indication for USO), age at first delivery (never, ≤30 years, 31–40 years, >40 years), family history of breast and ovarian cancer (yes/no), parity (0, 1, 2, and ≥3), and hysterectomy (yes/no). The “age at first delivery” was based on data from the Swedish Multi-generation Register, which includes information on all individuals born in 1932 or later. It contains detailed data on family relationships, including the biological parents, siblings and colleagues, which allows us to accurately determine the age at first delivery for individuals within the register. Comorbidity is an important factor affecting the health condition and risk of cancer. To account for this, we calculated the CCI, a validated tool that quantifies the burden of comorbidities using 17 weighted categories, including cardiovascular, and metabolic conditions and colleagues [26,27].

### Assessment of outcomes

We further linked these individuals to the Swedish Cancer Registry to identify patients who had been diagnosed with ovarian cancers using ICD-10 code C56 and fallopian tube cancers using ICD-10 code C57. Additionally, we identified patients from the Swedish National Patient Register who were diagnosed with CAD using the ICD-10 codes I21-I25 and with osteoporosis using ICD-10 codes M80 and M81. By further linking to the Cause of Death Register, we could identify individuals who had died during the follow-up period.

### Statistical analysis

We initially calculated the propensity score for undergoing USO using logistic regression with a consideration of date of birth, birth country, education level, region of residence, income, obesity, COPD, CCI, history of endometriosis, age at first delivery, and family history of breast or ovarian cancer. Subsequently, we matched women who underwent USO with those who did not, based on their propensity scores [28]. A caliper of less than 0.02 was applied for propensity score matching (PSM) in the study. The differences in demographic and clinical characteristics between the two groups were compared using the standardized mean difference (SMD). The two groups were considered an adequate balance if SMD was less than 0.1 [29]. Cox regression models were used to calculate hazard ratios (HRs) and 95% confidence intervals (CIs) of ovarian cancer, CAD, and osteoporosis associated with USO. The proportional hazard assumption was deemed reasonable by inspecting the Schoenfeld residuals. For women who received USO, the time of follow-up started on the date when USO was recorded (index date) and ended at the first date of diagnosis of specific outcomes, the date of death from any cause, and the end of the study period (31st December 2018), whichever came first. For the matched controls, follow-up began on the index date of their corresponding cases and continued until the occurrence of the earliest event listed above and USO. The final multivariable model was adjusted for age, birth country, education level, region of residence, income, obesity, COPD, CCI, endometriosis, age at first delivery, family history of ovarian and breast cancer, parity and hysterectomy. The association between USO and the long-term outcomes was stratified by hysterectomy. To assess the potential impact of unmeasured confounders on nullifying the observed exposure-outcome associations, E-values were calculated in our study [30].

Additionally, we have calculated the risk of CAD and osteoporosis among women with BSO as compared to women with USO using multivariate Cox regression and adjusted for date of birth, birth country, education level, region of residence, income, obesity, COPD, CCI, endometriosis, age at first delivery, family history of ovarian and breast cancer, parity and hysterectomy. All analyses were conducted using SAS version 9.4 (SAS Institute, Cary, NC).

## Results

### Study population

Fig 1 shows the flowchart of the study design. A total of 42,306 patients with USO were retrieved from the database. The demographic and clinical characteristics factors are shown in Table 1. The differences in baseline characteristics between women with USO and the matched controls were minimal, as indicated by SMD below the threshold of 0.1. All the variables listed in Table 1, along with parity and hysterectomy status, were adjusted in the final multivariable regression model. The main indications for USO are presented in S1 Table. The five most common indications included: (1) noninflammatory disorders of the ovary, fallopian tube and broad ligament (25.56%); (2) benign neoplasm of the ovary (24.66%); (3) leiomyoma of the uterus (12.87%); (4) endometriosis (10.34%); (5) inflammatory disease of ovary, fallopian tube, pelvic cellular tissue and peritoneum (5.97%).

**Table 1:** Baseline demographic and clinical characteristics between women with USO and matched controls.

	USO (*n* = 42,306)		Matched controls (*n* = 211,530)		SMD
	Mean	SD	Mean	SD	
Date of birth	43.77	11.07	43.77	10.89	0.013
	*N*	%	*N*	%	
Birth country					0.004
Sweden	35,374	83.6	178,373	84.3	
others	6,932	16.4	33,157	15.7	
Education					0.001
1–9	5,799	13.7	28,703	13.6	
10–11	19,771	46.7	99,064	46.8	
12+	16,736	39.6	83,763	39.6	
Region of residence					<0.001
Big city	6,538	15.5	31,997	15.1	
South	27,456	64.9	138,411	65.5	
North	8,311	19.6	41,117	19.4	
Unknown	1	0.0	5	0.0	
Income					0.005
Lowest	11,969	28.3	59,847	28.3	
Middle-low	10,945	25.9	54,382	25.7	
Middle-high	9,575	22.6	47,941	22.7	
Highest	9,817	23.2	49,360	23.3	
Obesity					0.014
No	41,312	97.7	206,845	97.8	
Yes	994	2.3	4,685	2.2	
COPD					0.014
No	39,836	94.2	199,532	94.3	
Yes	2,470	5.8	11,998	5.7	
CCI					0.014
0	37,546	88.7	188,349	89.0	
1	3,901	9.2	19,373	9.2	
2	614	1.5	2,736	1.3	
3+	245	0.6	1,072	0.5	
Endometriosis					0.053
No	33,466	79.1	167,861	79.4	
Yes	8,840	20.9	43,669	20.6	
Age at first delivery					0.014
Never	11,028	26.1	52,564	24.8	
≤30	26,755	63.2	136,224	64.4	
31–40	4,318	10.2	21,785	10.3	
>40	205	0.5	957	0.5	
Family history of breast cancer					0.013
No	38,138	90.1	191,062	90.3	
Yes	4,168	9.9	20,468	9.7	
Family history of ovarian cancer					0.008
No	41,791	98.8	209,247	98.9	
Yes	515	1.2	2,283	1.1	
Hysterectomy					
No	36,260	85.7	204,618	96.7	
Yes	6,046	14.3	6,012	3.3	
Parity					0.049
0	8,826	20.9	41,681	19.7	
1	7,194	17.0	32,981	15.6	
2	16,228	38.4	82,279	38.9	
≥3	10,058	23.7	54,589	25.8	

USO, unilateral salpingo-oophorectomy; SD, standard deviation; SMD, standardized mean difference; Education: total years of formal education completed; Income: individuals being categorized into quartiles based on their disposable income (Q1: lowest 25%, Q4: highest 25%); Obesity: body mass index ≥30 kg/m^2^; COPD, chronic obstructive pulmonary disease; CCI, Charlson comorbidity index.

### Risk of overall and histology-specific ovarian cancer

**Fig 2:**
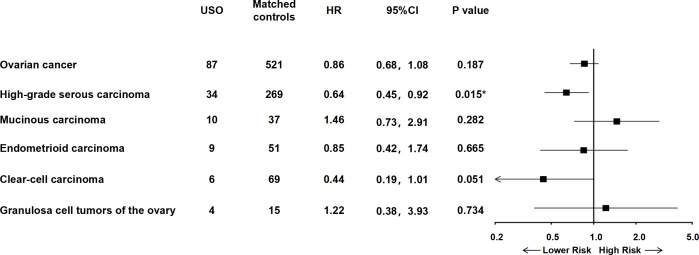
HR and 95% CIs of overall and histology-specific ovarian cancer associated with USO. Abbreviations: USO, unilateral salpingo-oophorectomy; HR, hazard ratios; CI, confidence interval.

The median follow-up time was 13 years (interquartile range: 6.58, 19.33). Associations between USO and ovarian cancer risk are shown in Fig 2 and S2 Table. After an accumulated 535,633 years of follow-up, the incidence rate of ovarian cancer among women with USO was 1.62 per 10,000 person-years, while the incidence rate for the matched controls was 1.89 per 10,000 person-years. USO demonstrated a non-significant reduced risk of ovarian cancer, with an adjusted HR of 0.86 (95% CI [0.68, 1.08]). When stratified by pathological type of ovarian cancer, a significantly negative association was observed for HGSCs, with an HR of 0.64 (95% CI [0.45, 0.92]). The *E*-value for the association between USO and HGSCs was 2.50 (confidence limit: 1.39). These values suggest that unmeasured confounding does not fully explain the association. When stratified by hysterectomy, no significant differences were found between USO and the risk of ovarian cancer (S2 and S3 Tables).

### Risk of CAD and osteoporosis

After a cumulative follow-up of 553,484 person-years, 1,008 women with USO were diagnosed with CAD (Table 2) with an incidence rate of 18.21 per 10,000 person-years. For osteoporosis, the incidence rate was 11.87 per 10,000 person-years among women with USO. Compared to women without USO, the HR of CAD among women with USO was 1.02 (95% CI [0.95, 1.09]). When stratified by hysterectomy, no significant differences were found between USO and the risk of CAD (S4 Table). Additionally, there was no association between USO and osteoporosis (HR = 1.06, 95% CI [0.98, 1.16]).

**Table 2:** Association of USO with CAD and osteoporosis.

	No. of individuals	Follow-up years	No. of outcome	IR	Crude			Adjusted*		
					HR	95% CI	*P*-value	HR	95% CI	*P*-value
CAD										
Matched controls	211,530	2,784,738	4,982	17.89	1			1		
USO	42,306	553,484	1,008	18.21	1.01	0.94, 1.08	0.832	1.02	0.95, 1.09	0.296
Osteoporosis										
Matched controls	211,530	2,792,807	3,090	11.06	1			1		
USO	42,306	554,569	658	11.87	1.07	0.98, 1.16	0.119	1.06	0.98, 1.16	0.153

Abbreviations: USO, unilateral salpingo-oophorectomy; CAD, coronary artery disease; No., Number; IR, incidence rate; HR, hazard ratio; CI, confidence interval.

Model adjusted for date of birth, birth country, education, region of residence, income, COPD, obesity, age at first delivery, CCI, history of endometriosis, family history of breast cancer, family history of ovarian cancer, and parity.

After excluding women with malignant tumors, a total of 4,922 women were identified from the National Patient Register with BSO before the age of 50. Among them, 123 women developed CAD and 68 developed osteoporosis during the follow-up period. Compared with women who had USO, the risk of CAD and osteoporosis showed no association with BSO, with HR of 1.18 (95% CI [0.97, 1.42]) and 1.04 (95% CI [0.81, 1.34]), respectively (S6 Table).

## Discussion

This population-based study, based on Swedish national registry data, aims to explore the association between USO and the long-term risk of ovarian cancer, CAD, and osteoporosis. We found that women who underwent USO before the age of 50 have a similar risk of CAD and osteoporosis as compared to the general population, suggesting that women who had USO did not encounter the adverse health outcomes typically associated with BSO. Additionally, our study indicated a reduced risk of HGSCs among women with USO. These results collectively propose that USO may serve as a potential strategy for decreasing the risk of ovarian cancer without resulting in CAD and osteoporosis.

The origin of HGSCs is now well-established, with the vast majority of cases arising from the fallopian tube [8,31,32]. Endometrioid and clear-cell ovarian cancers are generally considered to originate in areas affected by endometriosis [31]. Numerous studies have shown that prophylactic bilateral salpingectomy plays an important role in preventing HGSCs [33]. However, its effectiveness in preventing other histological types of ovarian cancer (such as endometrioid and clear cell cancers) is limited because these histological specific cancers arise in areas affected by endometriosis and are not primarily related to the fallopian tubes. Besides, the etiology of mucinous and low-grade serous ovarian cancers remains less clear and warrants further exploration. Exploring the association between USO and histology-specific ovarian cancer could offer additional evidence regarding the origin of specific types of ovarian cancer. Our study found a significantly lower risk of HGSCs which is consistent with the evidence from studies about the reduced risk of HGSCs among women who received prophylactic salpingectomy [34,35], supporting the theory that HGSCs is derived from the fallopian tube. In addition, clear-cell ovarian carcinoma showed a borderline reduced risk of 0.44. Given that endometriosis was a common indication for USO in our cohort, removing endometriotic tissue during surgery may explain the observed reduction in clear-cell carcinoma after USO. Additionally, the results were not statistically significant when we stratified the analysis by hysterectomy status, primarily due to the limited number of outcomes after stratification. However, other specific types of ovarian cancer did not show a statistically significant difference, suggesting that the origin of these specific types of ovarian cancer might need additional studies.

Currently, proponents of prophylactic BSO emphasize the significance of reducing the risk of ovarian cancer in women without fertility requirements [36,37]. However, many population-based studies have consistently demonstrated an association between BSO and cardiovascular disease. A meta-analysis reported a pooled estimate for the relative risk of cardiovascular disease among women who underwent bilateral oophorectomy compared to premenopausal women of 2.62 (95% CI [2.05, 3.35]). In addition, if stratified by the type of menopause, the pooled effect of bilateral oophorectomy on CAD was increased to 4.55 (95% CI [2.56, 8.01]), which differed from that of natural menopause [HR = 1.27, (95% CI [1.14, 1.43])] [38]. Furthermore, bone loss is also a concern following premature surgical menopause, and multiple studies have reported a significantly increased risk of osteoporosis after prophylactic BSO [39,40]. Compared to women who underwent surgical menopause after the age of 45 years, the risk of bone fracture was 3.64 times higher (95% CI [1.01, 13.04]) among women who had the surgery under the age of 45 [41]. Additionally, this association has also been observed in postmenopausal women [42]. Therefore, the pros and cons of prophylactic BSO should be carefully weighed [43]. However, there are few reports on studies of USO for health conditions such as cardiovascular disease and osteoporosis. A recent study noted that hysterectomy combined with unilateral oophorectomy showed an increased risk of cardiovascular disease, with an adjusted HR of 1.40 (95% CI [1.08, 1.82]) [44]. Surprisingly, this study suggested that BSO was associated with a modestly increased risk of cardiovascular disease compared to USO (adjusted HR 1.18), which contradicts the expected biological phenomenon. This discrepancy may indicate potential bias due to the limited number of cardiovascular outcomes in the cohort, highlighting the need for larger studies to validate these findings. In our study, we found that USO was not associated with CAD or osteoporosis.

To prevent the risk of ovarian cancer, NCCN guidelines recommend risk-reducing BSO at the appropriate age for women at high risk, such as those with BRCA gene mutation [45]. However, for premenopausal women, while a risk-reducing BSO can reduce the risk of specific ovarian cancer, it also increases the risk of cardiovascular health, bone health, cognitive health, and overall death, etc. [46]. Our study found that USO before age 50 reduced the risk of ovarian cancer in specific pathological types without adding health problems such as cardiovascular diseases and osteoporosis. This evidence can guide clinicians and provide more opportunities for surgical choice.

The advantage of this study is that it is based on nationwide registers in Sweden. The large sample size ensures the representativeness and statistical power of the overall Swedish population. By continuously updating the collected data, recall bias can be effectively eliminated and selection bias can be minimized. Registry-based data also provides us with information about underlying demographics and clinical confounders. Our matching criteria ensure similarity between comparison groups. Additionally, our study had an extended follow-up period of up to 26 years. The observed reduction in HGSCs risk remained consistent throughout the entire follow-up window. This prolonged temporal pattern effectively rules out detection bias arising from intraoperative visual inspection of the contralateral ovary during USO, as such evaluation would only account for short-term risk reduction of HGSCs. There are several limitations worth considering. First, the baseline characteristics lack some confounding factors for ovarian cancer, such as BRCA1/2 pathological mutant status, which might influence the outcome. However, we have adjusted for a family history of breast and ovarian cancers, which might partly minimize the confounding from genetic predisposition. Second, we also acknowledge the limitation of some potential covariables that we were unable to adjust, such as contraceptive use or breastfeeding, nutrient status, alcohol intake, and smoking. However, educational status and obesity, which are closely related to lifestyle and nutritional factors, were accounted for in our study. Additionally, COPD was also adjusted as a proxy for smoking in the current study. In sensitivity analyses, we calculated the E-value to assess the potential impact of unmeasured confounding. The results suggest that a confounder would need to exhibit strong associations (RR = 2.50) with both the exposure and outcome to fully negate the observed relationship. However, confounders with strong association (such as parity, family history and colleagues) were adjusted in our analyses, suggesting unmeasured confounding factors could not fully explain the observed association.

In conclusion, this population-based cohort study suggests that USO reduces the risk of HGSCs but was not associated with CAD or osteoporosis after a median 13-year follow-up.

## Supporting information

S1 RECORD Checklist Checklist of items, extended from the RECORD statement, that should be reported in observational studies using routinely collected health data. (DOCX)

S1 Fig Covariate balance before and after propensity score matching. Abbreviations: COPD, chronic obstructive pulmonary disease; CCI, Charlson comorbidity index.
(TIF)

S1 Table Underlying diseases among women with USO. Abbreviations: USO, unilateral salpingo-oophorectomy.
(DOCX)

S2 Table The crude and adjusted association of USO with ovarian cancer. Model 1 adjusted for date of birth, birth country, education, region of residence, income, COPD, obesity, age at first delivery, CCI, history of endometriosis, family history of breast cancer, family history of ovarian cancer and parity. Model 2 adjusted for variables in model 1 and hysterectomy. Abbreviations: USO, unilateral salpingo-oophorectomy; No., Number; IR, incidence rate; HR, hazard ratio; CI, confidence interval; HGSCs, high-grade serous carcinomas; COPD, chronic obstructive pulmonary disease; CCI, Charlson comorbidity index.
(DOCX)

S3 Table Association of USO with ovarian cancer stratified by hysterectomy. Abbreviations: IR, incidence rate; HR, hazard ratio; CI, confidence interval; USO, unilateral salpingo-oophorectomy; COPD, chronic obstructive pulmonary disease; CCI, Charlson comorbidity index.
(DOCX)

S4 Table Associations of USO with CAD stratified by hysterectomy. Abbreviations: IR, incidence rate; HR, hazard ratio; CI, confidence interval; USO, unilateral salpingo-oophorectomy; COPD, chronic obstructive pulmonary disease; CCI, Charlson comorbidity index.
(DOCX)

S5 Table Baseline demographic and clinical characteristics for women with BSO. Abbreviations: BSO, bilateral salpingo-oophorectomy; SD, standard deviation; COPD, chronic obstructive pulmonary disease; CCI, Charlson comorbidity index.
(DOCX)

S6 Table Associations of BSO with CAD and osteoporosis. Adjusted for date of birth, birth country, education, region of residence, income, COPD, obesity, age at first delivery, CCI, history of endometriosis, family history of breast cancer, family history of ovarian cancer and parity. Abbreviations: USO, unilateral salpingo-oophorectomy; BSO, bilateral salpingo-oophorectomy; CAD, coronary artery disease; No., Number; IR, incidence rate; HR, hazard ratio; CI, confidence interval; COPD, chronic obstructive pulmonary disease; CCI, Charlson comorbidity index.
(DOCX)

## Abbreviavtions

BSO: bilateral salpingo-oophorectomy
CAD: coronary artery disease
CCI: Charlson comorbidity index
CI: confidence interval
COPD: chronic obstructive pulmonary disease
HGSCs: high-grade serous carcinomas
HR: hazard ratio
ICD: International Classification of Diseases
IR: incidence rate
No.: Number
PSM: propensity score matching
RECORD: REporting of studies Conducted using Observational Routinely collected health Data
SD: standard deviation
SMD: standardized mean difference
USO: unilateral salpingo-oophorectomy
