# Supplementary figures and images for: Long-term outcomes after unilateral salpingo-oophorectomy: A registry-based retrospective cohort study
Source: PLoS Med. 2025 Jul 7;22(7):e1004639. doi: 10.1371/journal.pmed.1004639 (PMC12233271; doi:10.1371/journal.pmed.1004639)

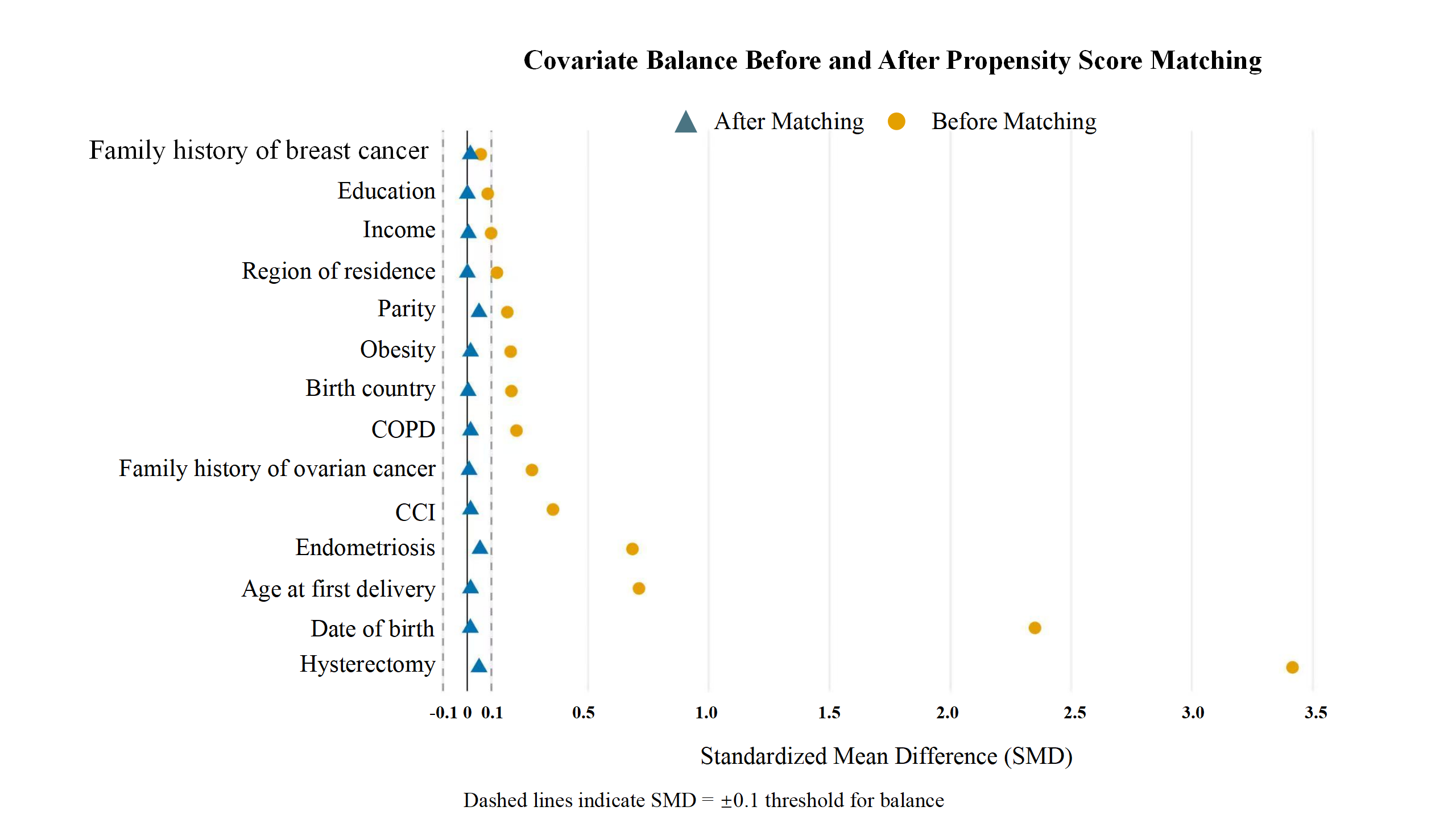

Supplement: S1 Fig — Abbreviations: COPD, chronic obstructive pulmonary disease; CCI, Charlson comorbidity index. (TIF) [file pmed.1004639.s002.tif]
