# Supplementary material for: Long-term outcomes after unilateral salpingo-oophorectomy: A registry-based retrospective cohort study
Source: PLoS Med. 2025 Jul 7;22(7):e1004639. doi: 10.1371/journal.pmed.1004639 (PMC12233271; doi:10.1371/journal.pmed.1004639)
Supplement: S1 Table — Abbreviations: USO, unilateral salpingo-oophorectomy. (DOCX) [file pmed.1004639.s003.docx]

**Supplementary Table 1.**

Underlying diseases among women with USO.

| The main indication for USO | Number | Percentage（%） |
| --- | --- | --- |
|  |  |  |
| Noninflammatory disorders of the ovary, fallopian tube and broad ligament | 10815 | 25.56 |
| Benign neoplasm of ovary | 10434 | 24.66 |
| Leiomyoma of uterus | 5443 | 12.87 |
| Endometriosis | 4374 | 10.34 |
| Inflammatory disease of the ovary, fallopian tube, pelvic cellular tissue and peritoneum | 2525 | 5.97 |
| Other female pelvic inflammatory diseases | 356 | 0.84 |
| Ectopic pregnancy | 325 | 0.77 |
| Excessive frequent and irregular menstruation | 192 | 0.45 |
| Disorders of the uterus, not elsewhere classified | 33 | 0.08 |
| Inflammatory disease of the uterus, except cervix | 20 | 0.05 |
| Inflammatory disease of cervix uteri | 17 | 0.04 |
| Others | 7772 | 18.37 |

Abbreviations: USO, unilateral salpingo-oophorectomy.
