# Supplementary material for: Long-term outcomes after unilateral salpingo-oophorectomy: A registry-based retrospective cohort study
Source: PLoS Med. 2025 Jul 7;22(7):e1004639. doi: 10.1371/journal.pmed.1004639 (PMC12233271; doi:10.1371/journal.pmed.1004639)
Supplement: S2 Table — Model 1 adjusted for date of birth, birth country, education, region of residence, income, COPD, obesity, age at first delivery, CCI, history of endometriosis, family history of breast cancer, family history of ovarian cancer and parity. Model 2 adjusted for variables in model 1 and hysterectomy. Abbreviations: USO, unilateral salpingo-oophorectomy; No., Number; IR, incidence rate; HR, hazard ratio; CI, confidence interval; HGSCs, high-grade serous carcinomas; COPD, chronic obstructive pulmonary disease; CCI, Charlson comorbidity index. (DOCX) [file pmed.1004639.s004.docx]

**Supplementary Table 2.**

The crude and adjusted association of USO with ovarian cancer.

| Histological-  specific ovarian cancer | No. of individuals | Follow-up years | No. of outcome | IR | Crude | | | Model 1 | | | Model 2 | | |
| --- | --- | --- | --- | --- | --- | --- | --- | --- | --- | --- | --- | --- | --- |
|  |  |  |  |  | HR | 95% CI | P value | HR | 95% CI | P value | HR | 95% CI | P value |
| Ovarian cancer |  |  |  |  |  |  |  |  |  |  |  |  |  |
| Matched controls | 211530 | 2762680 | 521 | 1.89 | 1 |  |  | 1 |  |  | 1 |  |  |
| USO | 42306 | 535633 | 87 | 1.62 | 0.87 | 0.69, 1.09 | 0.214 | 0.86 | 0.68, 1.08 | 0.187 | 0.88 | 0.70, 1.11 | 0.279 |
| HGSCs |  |  |  |  |  |  |  |  |  |  |  |  |  |
| Matched controls | 211530 | 2762680 | 269 | 0.97 | 1 |  |  | 1 |  |  | 1 |  |  |
| USO | 42306 | 535633 | 34 | 0.63 | 0.65 | 0.46, 0.90 | 0.011 | 0.64 | 0.45, 0.92 | 0.015 | 0.63 | 0.43, 0.92 | 0.018 |
| Mucinous |  |  |  |  |  |  |  |  |  |  |  |  |  |
| Matched controls | 211530 | 2762680 | 37 | 0.13 | 1 |  |  | 1 |  |  | 1 |  |  |
| USO | 42306 | 535633 | 10 | 0.19 | 1.47 | 0.74, 2.93 | 0.278 | 1.46 | 0.73, 2.91 | 0.282 | 1.70 | 0.80, 3.60 | 0.169 |
| Endometrioid |  |  |  |  |  |  |  |  |  |  |  |  |  |
| Matched controls | 211530 | 2762680 | 51 | 0.18 | 1 |  |  | 1 |  |  | 1 |  |  |
| USO | 42306 | 535633 | 9 | 0.17 | 0.90 | 0.44, 1.84 | 0.767 | 0.85 | 0.42, 1.74 | 0.665 | 0.95 | 0.47, 1.92 | 0.883 |
| Clear-cell |  |  |  |  |  |  |  |  |  |  |  |  |  |
| Matched controls | 211530 | 2762680 | 69 | 0.25 | 1 |  |  | 1 |  |  | 1 |  |  |
| USO | 42306 | 535633 | 6 | 0.11 | 0.44 | 0.19, 1.03 | 0.057 | 0.44 | 0.19, 1.01 | 0.051 | 0.48 | 0.21, 1.11 | 0.087 |
| Granulosa cell |  |  |  |  |  |  |  |  |  |  |  |  |  |
| Matched controls | 211530 | 2762680 | 15 | 0.05 | 1 |  |  | 1 |  |  | 1 |  |  |
| USO | 42306 | 535633 | 4 | 0.07 | 1.27 | 0.43, 3.77 | 0.668 | 1.22 | 0.38, 3.93 | 0.734 | 1.48 | 0.49, 4.45 | 0.489 |
| Others |  |  |  |  |  |  |  |  |  |  |  |  |  |
| Matched controls | 211530 | 2762680 | 80 | 0.29 | 1 |  |  | 1 |  |  | 1 |  |  |
| USO | 42306 | 535633 | 24 | 0.45 | 1.67 | 1.05, 2.66 | 0.031 | 1.77 | 1.11, 2.85 | 0.017 | 1.62 | 1.02, 2.58 | 0.042 |

Model 1 adjusted date of birth, birth country, education, region of residence, income, COPD, obesity, age at first delivery, CCI, history of endometriosis, family history of breast cancer, family history of ovarian cancer and parity.

Model 2 adjusted for variables in model 1 and hysterectomy.

Abbreviations: USO, unilateral salpingo-oophorectomy; No., Number; IR, incidence rate; HR, hazard ratio; CI, confidence interval; HGSCs, high-grade serous carcinomas; COPD, chronic obstructive pulmonary disease; CCI, Charlson comorbidity index.
