# Supplementary material for: Long-term outcomes after unilateral salpingo-oophorectomy: A registry-based retrospective cohort study
Source: PLoS Med. 2025 Jul 7;22(7):e1004639. doi: 10.1371/journal.pmed.1004639 (PMC12233271; doi:10.1371/journal.pmed.1004639)
Supplement: S3 Table — Abbreviations: IR, incidence rate; HR, hazard ratio; CI, confidence interval; USO, unilateral salpingo-oophorectomy; COPD, chronic obstructive pulmonary disease; CCI, Charlson comorbidity index. (DOCX) [file pmed.1004639.s005.docx]

**Supplementary Table 3.**

Association of USO with ovarian cancer stratified by hysterectomy.

|  | No. of individuals | Follow-up years | No. of outcome | IR |  | | |
| --- | --- | --- | --- | --- | --- | --- | --- |
|  |  |  |  |  | HR | 95% CI | P value |
| Hysterectomy |  |  |  |  |  |  |  |
| Matched controls | 6912 | 61494 | 14 | 2.28 | 1 |  |  |
| USO | 6046 | 63394 | 11 | 1.74 | 1.30 | 0.17, 9.75 | 0.797 |
| Non-hysterectomy |  |  |  |  |  |  |  |
| Matched controls | 204618 | 2701186 | 507 | 1.88 | 1 |  |  |
| USO | 36260 | 472239 | 76 | 1.61 | 0.90 | 0.70, 1.51 | 0.400 |

Abbreviations: IR, incidence rate; HR, hazard ratio; CI, confidence interval; USO, unilateral salpingo-oophorectomy; COPD, chronic obstructive pulmonary disease; CCI, Charlson comorbidity index.
