# Supplementary material for: Long-term outcomes after unilateral salpingo-oophorectomy: A registry-based retrospective cohort study
Source: PLoS Med. 2025 Jul 7;22(7):e1004639. doi: 10.1371/journal.pmed.1004639 (PMC12233271; doi:10.1371/journal.pmed.1004639)
Supplement: S4 Table — Abbreviations: IR, incidence rate; HR, hazard ratio; CI, confidence interval; USO, unilateral salpingo-oophorectomy; COPD, chronic obstructive pulmonary disease; CCI, Charlson comorbidity index. (DOCX) [file pmed.1004639.s006.docx]

**Supplementary Table 4.**

Associations of USO with CAD stratified by hysterectomy.

|  | No. of individuals | Follow-up years | No. of outcome | IR |  | | | | |
| --- | --- | --- | --- | --- | --- | --- | --- | --- | --- |
|  |  |  |  |  | HR | | 95% CI | | P value |
| Hysterectomy |  |  |  |  |  |  | |  | |
| Yes |  |  |  |  |  |  | |  | |
| Matched controls | 6912 | 61410 | 204 | 3.32 | 1 |  | |  | |
| USO | 6046 | 64767 | 156 | 2.41 | 0.78 | 0.49, 1.24 | | 0.301 | |
| No |  |  |  |  |  |  | |  | |
| Matched controls | 204618 | 2723328 | 4778 | 1.74 | 1 |  | |  | |
| USO | 36260 | 488717 | 852 | 1.74 | 1.01 | 0.94, 1.09 | | 0.729 | |

Abbreviations: IR, incidence rate; HR, hazard ratio; CI, confidence interval; USO, unilateral salpingo-oophorectomy; COPD, chronic obstructive pulmonary disease; CCI, Charlson comorbidity index.
