# Supplementary material for: Long-term outcomes after unilateral salpingo-oophorectomy: A registry-based retrospective cohort study
Source: PLoS Med. 2025 Jul 7;22(7):e1004639. doi: 10.1371/journal.pmed.1004639 (PMC12233271; doi:10.1371/journal.pmed.1004639)
Supplement: S5 Table — Abbreviations: BSO, bilateral salpingo-oophorectomy; SD, standard deviation; COPD, chronic obstructive pulmonary disease; CCI, Charlson comorbidity index. (DOCX) [file pmed.1004639.s007.docx]

**Supplementary Table 5.**

Baseline demographic and clinical characteristics for BSO population

|  | BSO (n=4922) | |
| --- | --- | --- |
|  | Mean | SD |
| Date of birth | 43.74 | 7.19 |
|  |  |  |
|  | N | % |
| Birth country |  |  |
| Sweden | 4132 | 83.9 |
| others | 790 | 16.1 |
| Education |  |  |
| 1-9 | 772 | 15.7 |
| 10-11 | 2431 | 49.4 |
| 12+ | 1719 | 34.9 |
| Region of residencce | |  |
| Big city | 655 | 13.3 |
| South | 3237 | 65.8 |
| North | 1030 | 20.9 |
| Unknown | 0 | 0.0 |
| Income |  |  |
| Lowest | 1604 | 32.6 |
| Middle-low | 1253 | 25.5 |
| Middle-high | 1039 | 21.1 |
| Highest | 1026 | 20.8 |
| Obesity |  |  |
| No | 4674 | 95.0 |
| Yes | 248 | 5.0 |
| COPD |  |  |
| No | 4587 | 93.2 |
| Yes | 335 | 6.8 |
| CCI |  |  |
| 0 | 4287 | 87.1 |
| 1 | 516 | 10.5 |
| 2 | 92 | 1.9 |
| 3+ | 27 | 0.5 |
| Endometriosis |  |  |
| No | 3549 | 72.1 |
| Yes | 1373 | 27.9 |
| Age at first delivery | |  |
| Never | 1320 | 26.8 |
| <=30 | 3044 | 61.8 |
| 31-40 | 534 | 10.8 |
| >40 | 24 | 0.5 |
| Family history of brest cancer | | |
| No | 4238 | 86.1 |
| Yes | 684 | 13.9 |
| Family history of ovary cancer | | |
| No | 4635 | 94.2 |
| Yes | 287 | 5.8 |
| Parity |  |  |
| 0 | 1189 | 24.2 |
| 1 | 832 | 16.9 |
| 2 | 1801 | 36.6 |
| >=3 | 1100 | 22.3 |

Abbreviations: BSO, bilateral salpingo-oophorectomy; SD, standard deviation; COPD, chronic obstructive pulmonary disease; CCI, Charlson comorbidity index.
