# Supplementary material for: Long-term outcomes after unilateral salpingo-oophorectomy: A registry-based retrospective cohort study
Source: PLoS Med. 2025 Jul 7;22(7):e1004639. doi: 10.1371/journal.pmed.1004639 (PMC12233271; doi:10.1371/journal.pmed.1004639)
Supplement: S6 Table — Adjusted for date of birth, birth country, education, region of residence, income, COPD, obesity, age at first delivery, CCI, history of endometriosis, family history of breast cancer, family history of ovarian cancer and parity. Abbreviations: USO, unilateral salpingo-oophorectomy; BSO, bilateral salpingo-oophorectomy; CAD, coronary artery disease; No., Number; IR, incidence rate; HR, hazard ratio; CI, confidence interval; COPD, chronic obstructive pulmonary disease; CCI, Charlson comorbidity index. (DOCX) [file pmed.1004639.s008.docx]

**Supplementary Table 6.**

Associations of BSO with CAD and osteoporosis.

|  | No. of individuals | Follow-up years | No. of outcome | IR | Crude | | | Adjusted | | |
| --- | --- | --- | --- | --- | --- | --- | --- | --- | --- | --- |
|  |  |  |  |  | HR | 95% CI | P value | HR | 95% CI | P value |
| CAD |  |  |  |  |  |  |  |  |  |  |
| USO | 42306 | 553484 | 1008 | 18.21 | 1 |  |  | 1 |  |  |
| BSO | 4922 | 52800 | 123 | 23.30 | 1.23 | 1.02, 1.50 | 0.028 | 1.18 | 0.98, 1.43 | 0.084 |
| Osteoporosis |  |  |  |  |  |  |  |  |  |  |
| USO | 42306 | 554569 | 658 | 11.87 | 1 |  |  | 1 |  |  |
| BSO | 4922 | 53064 | 68 | 12.81 | 1.03 | 0.80, 1.32 | 0.836 | 1.03 | 0.80, 1.33 | 0.794 |

Adjusted for date of birth, birth country, education, region of residence, income, COPD, obesity, age at first delivery, CCI, history of endometriosis, family history of breast cancer, family history of ovarian cancer and parity.

Abbreviations: USO, unilateral salpingo-oophorectomy; BSO, bilateral salpingo-oophorectomy; CAD, coronary artery disease; No., Number; IR, incidence rate; HR, hazard ratio; CI, confidence interval; COPD, chronic obstructive pulmonary disease; CCI, Charlson comorbidity index.
